# Trace impurities in sodium phosphate influences the physiological activity of *Escherichia coli* in M9 minimal medium

**DOI:** 10.1038/s41598-023-44526-4

**Published:** 2023-10-13

**Authors:** Yuki Soma, Saki Tominaga, Kanako Tokito, Yuri Imado, Kosuke Naka, Taizo Hanai, Masatomo Takahashi, Yoshihiro Izumi, Takeshi Bamba

**Affiliations:** 1 Division of Metabolomics/Mass Spectrometry Center, Medical Research Center for High Depth Omics, Medical Institute of Bioregulation, Kyushu University, 3-1-1 Maidashi, Higashi-ku, Fukuoka 812-8582 Japan; 2 Laboratory for Synthetic Biology, Graduate School of Bioresource and Bioenvironmental Sciences, Kyushu University, 744 Motooka, Nishi-ku, Fukuoka W5-729, 819-0395 Japan; 3 Shimadzu Corporation, 1, Nishinokyo-Kuwabara-cho, Nakagyo-ku, Kyoto 604-8511 Japan

**Keywords:** Bacterial techniques and applications, Applied microbiology, Mass spectrometry, Metabolic engineering, Bacterial genes, Bacterial physiology

## Abstract

In the field of applied microbiology, reproducibility and experimental variability are important factors that influence both basic research as well as process development for industrial applications. Experimental reproducibility and accuracy depend not only on culture conditions such as temperature and aeration but also on raw materials and procedures used for media preparation. The M9 minimal medium is one of the most common synthetic media for culturing *Escherichia coli* and other bacteria. This synthetic medium can be used to observe and evaluate the physiological activity of microbes under minimal nutritional requirements and determine the limiting factor for the desired phenotype. Although one of the advantages using the M9 medium is that its composition can be modulated, it is difficult to control presence of trace components and impurities from the reagents for preparing this medium. Herein, we showed that trace ingredients present in the reagents used for M9 media preparation affect the bacterial physiological activities (e.g., cell growth, substrate consumption, and byproduct formation). Additionally, we systematically identified the trace ingredient that influenced phenotypic differences. Our results showed that the selection of reagents and accuracy during reagent preparation is important for experimental reproducibility in the field of bio-engineering and systems biology focused on the systematic and continuous development of biomolecular systems (e.g., biorefinery, metabolic engineering, and synthetic biology).

## Introduction

Naturally occurring as well as recombinant microbes have been employed in bioengineering for several processes, such as fermented food production^1^, biofuel and chemical production^2,3^, bioremediation^4,5^, and microbiome engineering^6^. The selection and development of the culture media are important for the evaluation of microbial physiology and to obtain microbes of the desired phenotype. The M9 minimal medium is one of the most common synthetic media used to cultivate *Escherichia coli* and other bacteria^7^. This medium contains inorganic sources of nitrogen (NH_4_Cl), phosphate (KH_2_PO_4_ and Na_2_HPO_4_), and sulphate (MgSO_4_), along with some other additives such as thiamine and trace metals. Furthermore, a sole carbon source (e.g., glucose, glycerol, acetate, etc.) is also added to the M9 medium. Since the M9 medium is a synthetic medium, it can be used to observe and evaluate the physiological activity of microbes under minimal nutritional conditions and determine the limiting factor for the desired phenotype. Previous studies on microbial production of chemicals and fuel have employed the M9 medium as the basal medium^8–13^. In synthetic biology, the M9 medium has been used to evaluate the dynamic characteristics of synthetic genetic circuits^14–21^. Furthermore, the M9 medium is suitable for performing stable isotope tracing experiments, such as ^13^C turnover analysis and ^13^C metabolic flux analysis (^13^C-MFA)^22–25^ since unexpected contamination of ^12^C metabolites from natural nutrients (e.g. tryptone, yeast extract, etc.) can be prevented. Additionally, the M9 medium containing U-^13^C_6_ glucose as the sole carbon source can be used to prepare a stable isotope-labelled internal standards mixture (SILIS)^26–28^. SILIS is essential for chromatography-mass spectrometry-based quantitative metabolome analysis using the stable isotope dilution method (SIDM)^29–31^. These studies suggest that the M9 medium can be utilised in diverse fields of microbiology.

There are several different M9 recipes available in the Cold Spring Harbor Protocols^7^, Helmholtz Zentrum München (https://www.helmholtz-muenchen.de/), SubtiWiki^32^, etc. One of the media components, disodium phosphate (Na_2_HPO_4_), is available in both anhydrous and hydrated forms, and it is up to the experimenter to decide which reagent to use. Since anhydrous and hydrated disodium phosphate have the same chemical properties once dissolved in water at the same concentration, either recipe theoretically yields the same M9 medium. However, some researchers have shown empirically that microbial growth changes when the reagents and/or water used to make the media are altered^33, 34^.

Herein, we compared the physiological activity of *E. coli* cultured on M9 media prepared using disodium phosphate of different grades and purity. Hydrated and anhydrous disodium phosphate of different grades were purchased from different sources to prepare the M9 media. Bacterial growth was evaluated on these media using standard techniques. Our results suggest that the purity of reagents used for preparing the M9 media might influence the growth of *E. coli*.

## Results and discussion

### *E. coli* growth on M9 media containing disodium phosphate anhydrous or different hydrated forms (dihydrate, heptahydrate, and dodecahydrate).

Disodium phosphate was purchased from two different companies [Nacalai Tesque Ltd. and Merck KGaA (Darmstadt, Germany)] in different forms and grades (Table 1). Na_2_HPO_4_ anhydrous (EP grade), Na_2_HPO_4_ · 7H_2_O (GR grade), and Na_2_HPO_4_ · 12H_2_O (EP grade) were purchased from Nacalai Tesque Ltd. while Na_2_HPO_4_ · 2H_2_O (analysis grade EMSURE) was purchased from Merck KGaA.

**Table 1:** Disodium phosphate reagents used in this study.

Identifier	Formula	Grade	Purity	Maker or provider
A	Na_2_HPO_4_	Extra pure reagent (EP)	≧ 98.0%, (T)	Nacalai Tesque, Ltd
B	Na_2_HPO_4_ · 2H_2_O	Merck EMSURE^®^	≧ 99.5%, (T)	E. Merck KG
C	Na_2_HPO_4_ · 7H_2_O	Guaranteed reagent (GR)	≧ 99.0%, (T)	Nacalai Tesque, Ltd
D	Na_2_HPO_4_ · 12H_2_O	Extra pure reagent (EP)	≧ 98.0%, (T)	Nacalai Tesque, Ltd
A (1G)	Na_2_HPO_4_	WAKO 1st grade	≧ 99.0%, (T)	FUJIFILM WAKO Chemicals, Corp
A (GR)	Na_2_HPO_4_	Guaranteed reagent (GR)	≧ 99.0%, (T)	FUJIFILM WAKO Chemicals, Corp
B	Na_2_HPO_4_ · 2H_2_O	Merck EMSURE^®^	≧ 99.5%, (T)	E. Merck KG
C (ACS)	Na_2_HPO_4_ · 7H_2_O	ACS reagent grade	98.0–102.0%	MP Biomedicals, Inc
D (1G)	Na_2_HPO_4_ · 12H_2_O	WAKO 1st grade	≧ 98.0%, (T)	FUJIFILM WAKO Chemicals, Corp
D (GR)	Na_2_HPO_4_ · 12H_2_O	Guaranteed reagent (GR)	≧ 99.0%, (T)	FUJIFILM WAKO Chemicals, Corp

**Figure 1:**
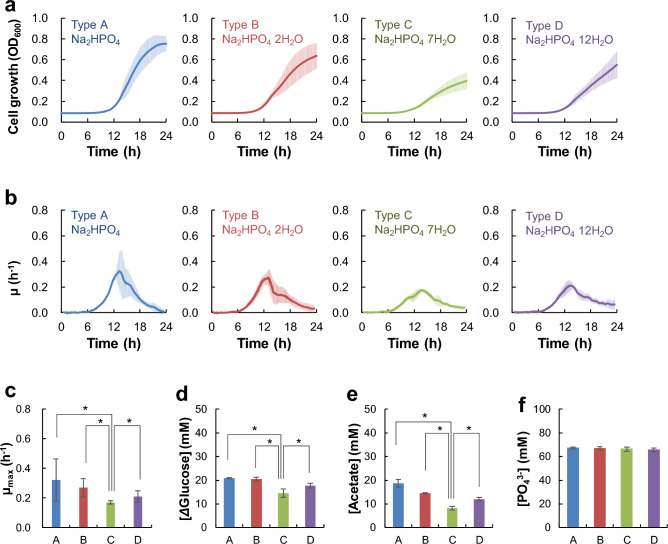
Physiological activity of *E. coli* in M9 media prepared using different disodium phosphate reagents. Four types of M9 media were prepared: Type A containing Na_2_HPO_4_ (EP grade), Type B containing Na_2_HPO_4_ · 2H_2_O (Analysis grade), Type C containing Na_2_HPO_4_ · 7H_2_O (ACS grade), and Type D containing Na_2_HPO_4_ · 12H_2_O (EP grade). (**a**) Bacterial cell growth in M9 media (2 g/L glucose) in 96 well plates at 37 °C and 269 rpm, (**b**) One-hour rolling average specific cell growth rate (μ), (**c**) Maximum specific cell growth rate (μ_max_), (**d**) Glucose consumption, (**e**) Acetate production, (**f**) Initial concertation of phosphate in each medium. Error bars indicate standard deviation. *p < 0.05 (Welch's *t*-test), *n* = 24.

Except for disodium phosphate, all other reagents used in four different types of M9 media (Type A, B, C, and D) were the same. These media were then used to culture *E. coli* strain BW25113 (Fig. 1). Although the same seed culture broth (overnight LB culture) was passaged with the same passage ratio (0.01% v/v) into each M9 medium, different cell growth profiles were observed in different M9 media (Fig. 1a, b). The time taken by the bacterial cells to enter the logarithmic growth phase (log phase) was not significantly different (approximately 750 min) among all M9 media, although the cell growth rate and final cell density were different (Fig. 1a). The final *E. coli* density in M9 type C (disodium phosphate heptahydrate) was lowest, and 51% lower than that observed in the M9 type A media. Furthermore, the specific growth rate (μ) of bacterial cells was also different in different M9 media (Fig. 1b). The maximum specific growth rate (μ_max_) in M9 type C was significantly lower than that in other M9 media (Fig. 1c). Glucose consumption and metabolic product (acetate) formation were lowest in M9 type C (Fig. 1d, e). Our HPLC analysis showed no significant difference in phosphate concentration of different M9 media used in this investigation (Fig. 1f).

Altogether, our results suggest that differences in cell growth and other physiological activities of *E. coli* observed in different M9 media are not dependent on the phosphate concentration, but are influenced by differences in disodium phosphate. The reproducibility of this experiment was confirmed through three different experiments (Fig. S1, *n* = 24 for each experiment). After 24 h of cultivation, approximately 6% of the total media was evaporated (Fig. S2a), and the evaporation volume varied among the 96 wells (RSD = 8.2%) (Fig. S2b–d). Evaporation of the media was especially considerable at the outer periphery of the plate. To investigate the influence of unequal media evaporation, the sample positions were replaced and the cell growth of *E. coli* on each M9 medium was monitored (Type A–D) (Fig. S3). Consequently, the growth efficiency on each medium was reproduced regardless of the sample position.

### Differences in disodium phosphate reagents affect the growth of multiple *E. coli* substrains under different environmental conditions

Next, we investigated the effects of environmental conditions, such as temperature, aeration, and carbon/nitrogen ratio (C/N ratio) on *E. coli* growth in different M9 media. Our results showed that altering the culture temperature (30 °C, 37 °C, and 42 °C) affects the bacterial cell growth in different M9 media (Fig. S4). *E. coli* growth was maximum at the optimum temperature (37 °C) (Fig. S4a–c). Cell growth at 30 °C was drastically suppressed (Fig. S4a), although the final cell density and the μ_max_ were the same in all four media (M9 types A–D) at this temperature (Fig. S4g). Similar results were observed at 42 °C (Fig. S4i). Therefore, the poor growth of *E. coli* cells in M9 type C was not temperature- or C/N ratio-dependent (Fig. S5), and aeration was regulated by shaking speed (Fig. S6). This was also observed during test tube cultivation (Fig. S7). Increasing the shaking speed and culturing in a test tube improved the oxygen supply in the media and promoted growth in all M9 media types, but poor growth was still observed in medium C.

Similar results were observed with other *E. coli* substrains, including K-12, B, and BL21(DE3) strains (Fig. S8). These results confirmed that differences in cell growth observed in different M9 media were due to variations in the disodium phosphate reagent independently of the *E. coli* strain and culture conditions. However, the presence of other impurities in the disodium phosphate reagent cannot be ruled out which could have affected the growth of *E. coli.*

**Figure 2:**
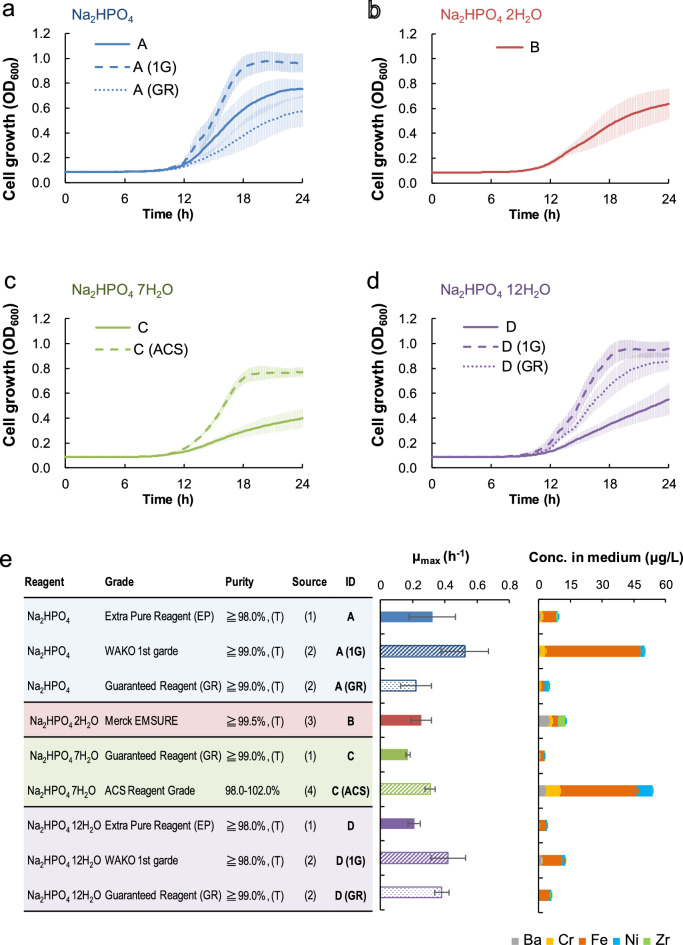
Effects of reagent grade of disodium phosphate for M9 medium preparation on bacterial cell growth. Bacterial cell growth on different M9 media are shown in (**a**)–(**d**). (**e**) The maximum specific growth rate (left panel) and amount of impurities (quantified by ICP-MS analysis; right panel) are shown with the Na_2_HPO_4_ reagents’ information. The amount of impurities are shown in stacked bars; Ba (grey), Cr (yellow), Fe (orange), Ni (blue), and Zr (green). Error bars indicate the standard deviation (*n* = 24).

Next, we evaluated *E. coli* growth on M9 media containing disodium phosphate of different grades. Disodium phosphate reagents were additionally obtained from FUJIFILM WAKO Chemicals, Corp. (Table 1). For Na_2_HPO_4_ and Na_2_HPO_4_ · 12H_2_O, both WAKO 1st grade (A (1G) and B (1G)) and GR grade (A (GR) and B (GR)) were obtained. Since Na_2_HPO_4_ · 2H_2_O was available only with E. Merck KG, we used this reagent (B). For Na_2_HPO_4_ · 7H_2_O, we obtained one ACS Grade reagent (C (ACS)) from MP Biomedicals, Inc. As shown in Fig. 2a–d, the growth of *E. coli* was significantly different in M9 media prepared using different disodium phosphate reagents. Cell growth in M9 type C (ACS) (prepared using Na_2_HPO_4_ · 7H_2_O from MP Biomedicals, Inc.) improved to the same extent as other M9 types (A, B, and D) (Fig. 2). In addition, variations in *E. coli* growth were observed among different M9 media prepared using different grade reagents from the same manufacturer (Fig. 2; A (1G) vs. A (GR)). The amount and purity of the water of hydration in all reagents of different grades were taken into consideration before preparing the media, therefore, the final concentration of phosphoric acid and sodium was constant. This suggests that any differences in these M9 media components must be due to the presence of impurities.

### Identification of impurities that affect bacterial cell growth

To investigate the impurities present in Na_2_HPO_4_ reagents that affect *E. coli* cell growth, we performed the elemental analysis of Na_2_HPO_4_ reagents using inductively coupled plasma mass spectrometry (ICP-MS). All Na_2_HPO_4_ reagents used in this study were analysed, and five trace impurities were detected (Ba, Cr, Fe, Ni, and Zr). As shown in Fig. 2e, the total amount of impurities varied among the reagents, and a positive correlation was found between the total amount of impurities and the maximum specific growth rate (μ_max_) (Fig. S9a). A positive correlation was also observed between the amount of iron present and the maximum specific growth rate (μ_max_) (Fig. S9d). Iron was detected in all the Na_2_HPO_4_ reagents (Table S9). The amounts of other impurities (Ba, Cr, Ni, and Zr) were not significantly correlated with growth rate (Fig. S9b, c, e, f). M9 type C contained the lowest amount of total impurities among the media ([total metals] = 4.1 μg/L), and the cell growth rate was also lowest in this medium (μ_max_ = 0.17 h^-1^) (Fig. 2e). M9 type C contained 2.22 ± 0.09 μg/L of iron, 0.09 ± 0.08 μg/L of barium, and 0.09 ± 0.08 μg/L of zirconium. The iron content was comparable with the media, which supported increased cell growth (M9 type B and D). Of the elements not detected in M9 type C (Cr and Ni), Ni was detected in all the other media. Additionally, Ni was not found in the A5 (Merck KGaA) used in this study.

**Figure 3:**
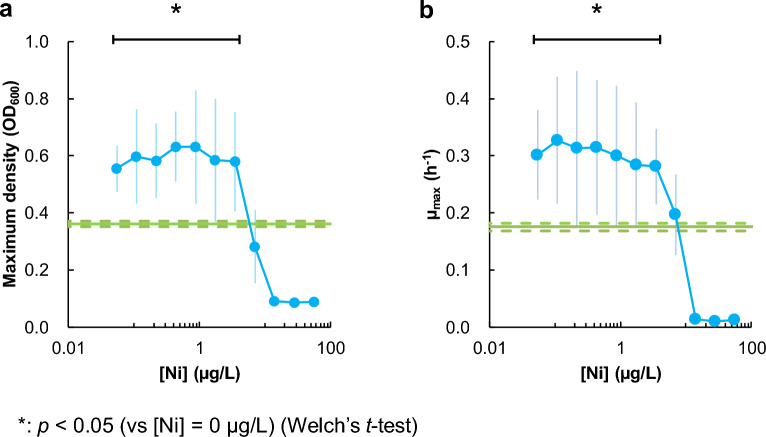
Effects of nickel supplementation in M9 medium type C on bacterial cell growth. (**a**) Maximum cell density after 24 h of shaken culture. (**b**) The maximum specific cell growth (μ_max_). Closed circles represent results at specific nickel concentrations. Green lines indicate results without nickel supplementation ([Ni] = 0 μg/L), and green dashed lines indicate the range of standard deviation. Error bars indicate the standard deviation. *p < 0.05 (Welch's *t*-test), *n* = 8.

To investigate the effect of Ni on bacterial cell growth, we then cultured *E. coli* in M9 Type C with various concentrations of nickel (Fig. 3). The final *E. coli* cell density after 24 h of shaking culture was 37–43% increased in the presence of nickel (NiSO_4_) in a specific concentration range (0.06–3.7 μg/L) (Fig. 3a). The maximum specific cell growth (μ_max_) was also increased (42–69%) under the same range of NiSO_4_ (Fig. 3b). In contrast, excess nickel (> 10 μg/L) significantly suppressed cell growth. These results confirm that nickel in a specific concentration range promotes *E. coli* growth.

### [NiFe] hydrogenases enhance the cell growth on M9 medium

**Figure 4:**
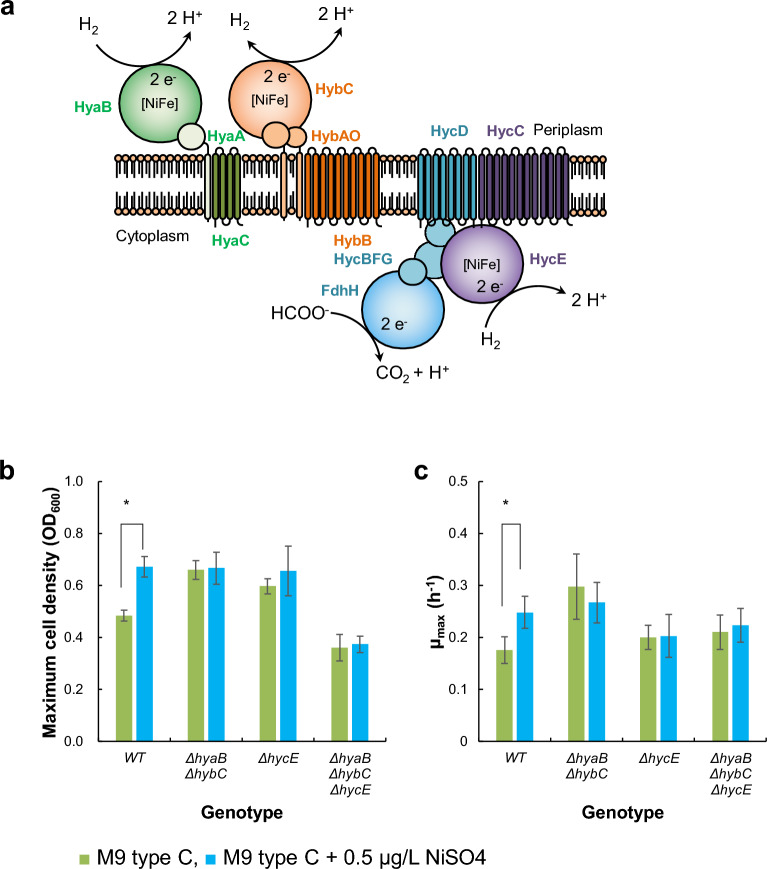
Deletion of genes encoding Ni-dependent enzymes counteracts the enhancement of cell growth by Ni supplementation in M9 type C. (**a**) Illustration showing *E. coli* [NiFe] hydrogenases. (**b**) Maximum cell density and (**c**) maximum specific cell growth rate (μ_max_) of various *E. coli* strains in 96-well plates cultured using M9 type C with (blue) or without (green) 0.5 µg/L NiSO_4_. Error bars indicate the standard deviation. *p < 0.05 (Welch's *t*-test), *n* = 8. Figures are original, created using Microsoft PowerPoint 2019 for Windows version 1808 (https://www.microsoft.com/).

Ni is known to play an important role in the biology of some eubacteria. In *E. coli*, [NiFe] hydrogenases (Hyd) are oxidative enzymes that catalyse the reversible oxidation of molecular hydrogen to protons and electrons^35,36^. Hydrogenases play an important role in energy metabolism in several microorganisms^37^. Three types of [NiFe] hydrogenases have been identified in *E. coli*: Hyd-1, Hyd-2, and Hyd-3 (Fig. 4a)^38^. Hyd-1 consists of three subunits (HyaA, HyaB, and HyaC) and is an oxygen-tolerant H_2_ oxidiser. Hyd-2 is an O_2_-sensitive [NiFe] hydrogenase involved in anaerobic respiration and consists of subunits HybA, HybB, HybC, and HybO. Hyd-3 is a part of the formate hydrogenlyase complex (FHL) that is produced under fermentative conditions and is required for the disproportionation of formate to CO_2_ and H_2_.

Several studies have reported that each Hyd is involved in the activation of F_O_F_1_-ATPase under different non-neutral pH conditions^39–41^. Moreover, Hyd enzymes may function as intracellular pH monitors through F_O_F_1_-ATPase which interacts with the FHL complex composed of FDH and Hyd to regulate the cytoplasmic pH^40^. According to these reports, nickel-dependent hydrogenases can support the growth of *E. coli* under microaerobic and acidic conditions by facilitating the homeostasis of intracellular energy/redox and pH. To investigate the relationship between these [NiFe] hydrogenases and the enhanced growth of *E. coli* in the presence of nickel sulfate, we prepared *E. coli* strains by deleting genes responsible for the activity of [NiFe] hydrogenases (*ΔhyaB*, *hybC*, *ΔhycE,* and *ΔhyaB*, *hybC*, *hycE*). When these strains were cultured in M9 type C with 0.05 µg/L of NiSO_4_, no significant enhancement of cell growth was observed (Figs. 4b, c and S10). These results clearly demonstrate that [NiFe] hydrogenases support the growth of *E. coli* on minimal media containing trace amounts of nickel. It is suggested that these enzymes regulate the physiological activity of *E. coli* by balancing the intracellular redox and energy metabolism in the presence of appropriate amounts of nickel and iron.

The selection of reagents and accuracy in reagent preparation is important for the reproducibility of systematic experiments in the field of bio-engineering, which requires systematic and continuous development of biosystems (e.g., biorefinery, metabolic engineering, and synthetic biology). Moreover, selection and preparation of culture media is important to systematically evaluate the effects of gene deletion or upregulation using common bioresource platforms such as bacterial strain libraries^42–44^. This study demonstrated that the physiological activity of *E. coli* is significantly affected by the trace impurities present in reagents, used in growth media. Trace impurities were supposed to affect enzyme activities of [NiFe] hydrogenase in *E. coli*. Further validation experiments, such as metabolic ^13^C metabolic flux analysis and enzyme activity assay for each [NiFe] hydrogenase, might be helpful to quantify the contribution of these impurity-induced changes of enzymatic activity to bacterial growth and energy metabolism.

## Conclusion

Herein, we demonstrated that bacterial physiological activity is affected by impurities present in the reagents used for medium preparation exemplified with Na_2_HPO_4_, even when we intended to prepare the same medium. Furthermore, Ni-dependent hydrogenases assist the growth of *E. coli* on M9 minimal medium containing glucose as the sole carbon source and a very small amount of nickel as an impurity. Slight differences in medium preparation (purity, grade, manufacturer, and manufacturing lot of reagents) should be considered when performing microbiological experiments to ensure consistency and reproducibility.

## Materials and methods

### Chemicals and reagents

All chemicals were purchased from Nacalai Tesque Ltd. (Kyoto, Japan) and Fujifilm Wako Pure Chemical Industry Ltd. (Osaka, Japan), unless otherwise specified. The trace metal solution A5 (H_3_BO_3_; 2860 mg/L, MnCl_2_ · 4H_2_O; 1810 mg/L, ZnSO_4_ · 7H_2_O; 222 mg/L, Na_2_MoO_4_ · 2H_2_O; 390 mg/L, CuSO_4_ · 5H_2_O; 79 mg/L, Co(NO_3_)_2_ · 6H_2_O; 49 mg/L) was purchased from Merk KGaA (Darmstadt, Germany).

### Preparation of the M9 medium

The 5 × M9 salts solutions were prepared using several different disodium phosphate reagents. Tables S5–S8 summarizes all the disodium phosphate and other reagents (KH_2_PO_4_, NaCl, and NH_4_Cl) used for each 5 × salts solution (Type A ~ D). The Each 5 × salts solution was used for preparation of M9 media (Type A ~ D) supplemented with 4 g/L glucose, 10 ppm thiamine hydrochloride, and 10 ppm trace metal solution A5.

### Bacterial strains and culture condition

*E. coli* strains BW25113, K-12, B, and BL21(DE3) were obtained from National BioResource Project (NBRP-E.coli at NIG), Japan. All strains were stored in 15% glycerol stocks at − 80 °C. Single-gene knockout variants of BW25113, JW0955 (*ΔhyaB*), JW2962 (*ΔhybC*), and JW2691 (*ΔhycE*) were obtained from the Keio collection^42^ and used for multiple [NiFe] hydrogenase genes deletion via P1 transduction^45^.

The glycerol stocks of each strain were inoculated into 5 mL of LB medium in 30-mL test tubes as the seed culture, which was then incubated at 37 °C with orbital shaking (250 rpm) overnight. The overnight grown seed culture was passaged into fresh M9 medium (0.01% v/v). Afterwards, 200 μL of this solution was dispensed into a 96-well plate, which was covered with a clear plastic lid without sealing to ensure adequate air supply. The covered plate was then incubated with orbital shaking in a multi-mode plate reader Synergy HTX (BioTek Instruments, Winooski, VT). To prevent fogging of the plate lid due to medium evaporation, cultivation was carried out in the condensation control mode of the plate reader. The cultivation temperature and orbital shaking conditions were set to 37°°C and 269 rpm (orbital rotation, φ = 6 mm) unless otherwise specified. The initial inoculum amount for the 96-well main culture was set so that the final cell density (OD_600_) did not exceed the detection limit of the plate reader (OD_600_ ≦ 0.99) after 24 h of cultivation (Fig. S11). Optical density at λ = 600 nm (OD_600_) was automatically measured every 10 min for 24 h.

### Quantification of phosphate, glucose, and organic acids

The extracellular glucose concentration was quantified using high-performance liquid chromatography (HPLC) on LC-20AD, SIL-20ACHT, CTO-20AC, and RID-10A instruments (Shimadzu Corp., Kyoto, Japan) equipped with a ligand-exchange chromatography column (ULTRON AF-HILIC-CD, 4.6 mm × 250 or 150 mm, 5 mm, Shimadzu Corp.). The samples were separated using an 85% acetonitrile aqueous solution. The column temperature was set to 60 °C and the flow rate was adjusted to 0.8 mL/min. Five microliter of each sample was injected.

Extracellular organic acids and phosphate were quantified using HPLC on LC-30AD, SIL-30AC, CTO-20AC, and CDD-10AVP instruments (Shimadzu Corp.) equipped with three tandem ion-exclusion chromatography columns (Shim-pack Fast-OA, 7.8 × 100 mm, 5 mm, Shimadzu Corp.) and a guard column (Shim-pack Fast-OA (G), 4.0 × 10 mm, 5 mm, Shimadzu Corp.). Sample separation was achieved using a 5.0 mM *p*-toluenesulfonate mobile phase. The column temperature was set at 40 °C and the flow rate was adjusted to 0.8 mL/min. CDD-10AVP was used as the detector for the post-column pH-buffered electrical conductivity. Post-column pH buffering was attained using a pH-buffering solution containing 5.0 mM *p*-toluenesulfonate, 20 mmol/L Bis–Tris, and 0.1 mmol/L EDTA. Five microliter of each sample was injected.

### Trace element analysis in four different disodium phosphate reagents using ICP-MS

Aqueous solutions of disodium phosphate (100 mg per 150 mL) were prepared for ICP-MS analysis using the following reagents: Na_2_HPO_4_ anhydrous (EP grade, Nacalai Tesque Ltd.), Na_2_HPO_4_ · 2H_2_O (Analysis grade EMSURE, Merck KGaA), Na_2_HPO_4_ · 7H_2_O (GR grade, Nacalai Tesque Ltd.), and Na_2_HPO_4_ · 12H_2_O (EP grade, Nacalai Tesque Ltd.). Result was normalised to the weight of the disodium phosphate reagent used for media preparation.

A calibration curve for each target ion was prepared using the following standard solutions: Nickel Standard Solution (Fujifilm Wako Pure Chemical) was diluted with 1% (v/v) HNO_3_ ([Ni] = 0, 0.02, 0.2 μg/L); Barium Standard Solution (Kanto Chemical Co., Inc., Tokyo, Japan) was diluted with 1% (v/v) HNO_3_ ([Ba] = 0, 0.1, 0.2, and 1 µg/L); Chromium Standard Solution (Fujifilm Wako Pure Chemical) was diluted with 1% (v/v) HNO_3_　([Cr] = 0, 0.1, 0.2, and 1 µg/L); Iron Standard Solution (Kanto Chemical Co., Inc.) was diluted with 1% (v/v) HNO_3_ ([Fe] = 0, 0.1, 0.2, and 1 µg/L); and Zirconium Standard Solution (Fujifilm Wako Pure Chemical) was diluted with 1% (v/v) HNO_3_ ([Zr] = 0, 1 µg/L).

ICPMS-2030 (Shimadzu Co.) was operated with the following conditions: RF frequency power, 1.20 kW; plasma gas flow, 9.0 L/min; auxiliary gas flow, 1.10 L/min; carrier gas 0.70 L/min; nebulizer, coaxial; sampling depth, 7.0 mm; cone material, copper; internal standard, automatic addition (T-piece); sample tube, black-black PVC (internal diameter 0.76 mm); internal standard tube, orange-blue PVC (internal diameter 0.25 mm).

### Statistical analysis and reproducibility of results

All the statistical analyses were performed using Microsoft Excel 2016. Data are shown as individual data points with mean ± standard deviation. Sample size has been mentioned in each figure and/or figure legend. All experiments presented in the manuscript were independently repeated at least two times with consistent results. The statistical significance of each experiment was determined by the Welch’s two-tailed *t*-test and a *p*-value less than 0.05 was considered statistically significant.

### Supplementary Information

Supplementary Information 1.

Supplementary Information 2.

## Data Availability

The authors declare that the source data supporting the findings of this study are available within the article and its supplementary information files (Supplementary Data 1). The other datasets generated during and/or analysed during the current study are available from the corresponding author upon reasonable request.
