# Supplementary material for: Trace impurities in sodium phosphate influences the physiological activity of Escherichia coli in M9 minimal medium
Source: Sci Rep. 2023 Oct 13;13:17396. doi: 10.1038/s41598-023-44526-4 (PMC10576033; doi:10.1038/s41598-023-44526-4)
Supplement: Supplementary file 1 — Supplementary Information 1. [file 41598_2023_44526_MOESM1_ESM.pdf]

Supplementary File

Trace impurities in sodium phosphate influences the physiological activity of Escherichia coli in M9 minimal medium.

Yuki Soma[1,2], Saki Tominaga[1], Kanako Tokito[1], Yuri Imado[1], Kosuke Naka[3], Taizo Hanai[2], Masatomo Takahashi[1], Yoshihiro Izumi[1], and Takeshi Bamba[1]*

[1]Division of Metabolomics/Mass Spectrometry Center, Medical Research Center for High Depth Omics, Medical Institute of Bioregulation, Kyushu University, 3-1-1 Maidashi, Higashi-ku, Fukuoka 812-8582, Japan

[2]Laboratory for Synthetic Biology, Graduate School of Bioresource and Bioenvironmental Sciences, Kyushu University, W5-729, 744 Motooka, Nishi-ku, Fukuoka 819-0395, Japan

[3]Shimadzu Corporation, 1, Nishinokyo-Kuwabara-cho, Nakagyo-ku, Kyoto 604-8511, Japan

[*] Corresponding author

TEL: +81-92-642-6171

FAX: +81-92-642-6172

E-mail: bamba@bioreg.kyushu-u.ac.jp

Supplementary figure

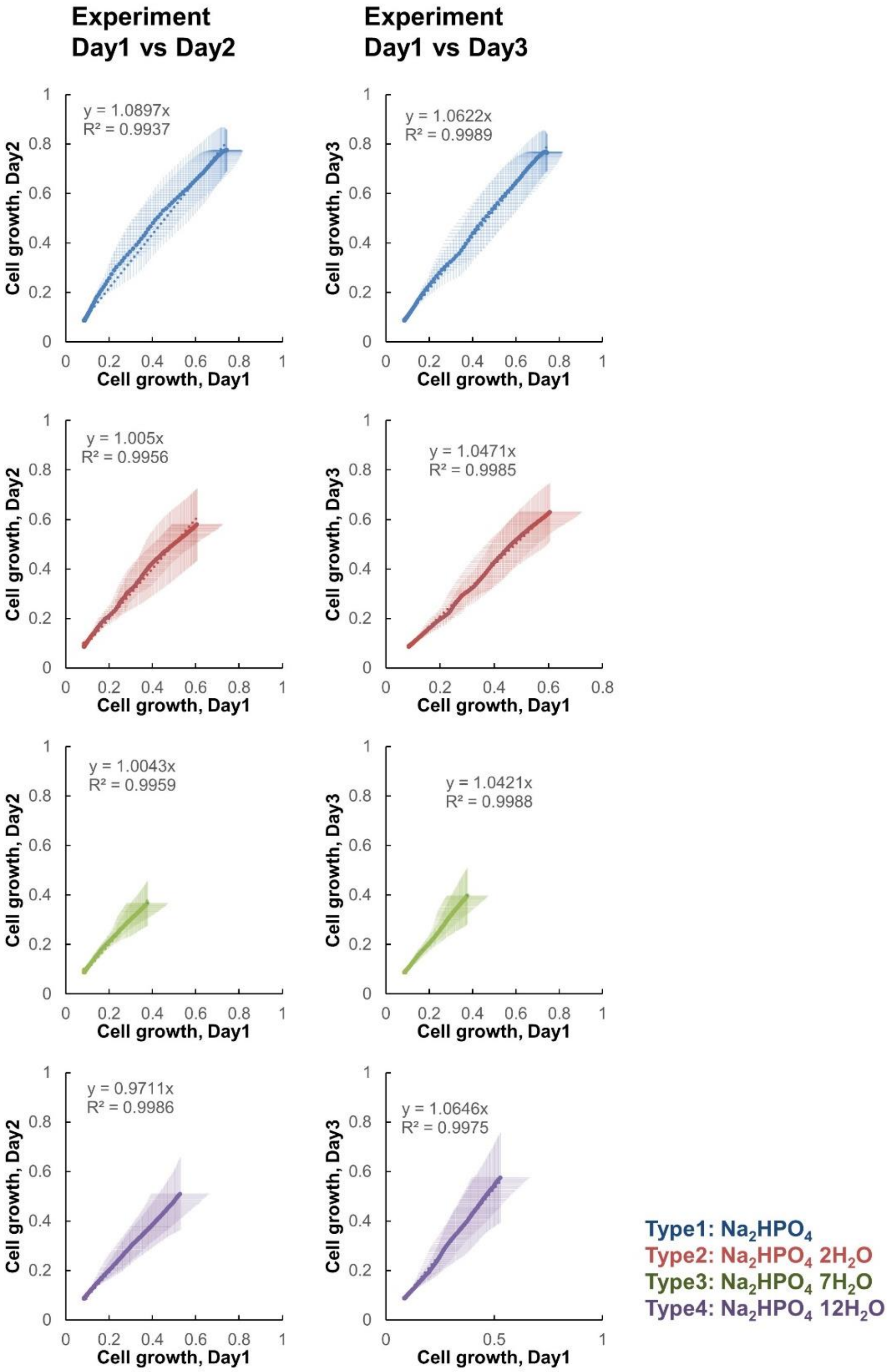

Fig. S1. Reproducibility of cultivation using different four type of M9 medium. Same cultivation was performed three times among three days. Each graph is plotting cell density at the same time point during the 96-well plate cultivation for the two data sets. Left panels show the comparison of experiment performed Day1 and Day2, right panels show the comparison of experiment performed Day1 and Day3. Error bars indicate standard deviation (n=24).

a

Evaporation amount

Initial plate weight (at 0 h)	= 81.1164 g (203.8 μL per well)
Final plate weight (at 24 h)	= 79.9181 g (191.2 μL per well)
*Δ*Weight of liquid (at 24 h)	= -1.19830 g (12.4 μL per well)
Evaporation volume (at 24 h)	= 6.1% of initial volume

b

	1	2	3	4	5	6	7	8	9	10	11	12	
A	0.6	0.564	0.568	0.574	0.566	0.566	0.578	0.592	0.588	0.583	0.576	0.553	A
B	0.57	0.588	0.583	0.566	0.565	0.577	0.571	0.572	0.574	0.578	0.578	0.582	B
C	0.553	0.575	0.557	0.571	0.556	0.553	0.543	0.558	0.551	0.556	0.54	0.558	C
D	0.568	0.567	0.563	0.572	0.578	0.582	0.569	0.558	0.571	0.574	0.571	0.59	D
E	0.583	0.574	0.567	0.569	0.561	0.569	0.571	0.573	0.572	0.562	0.557	0.558	E
F	0.577	0.558	0.558	0.563	0.574	0.57	0.571	0.577	0.569	0.573	0.589	0.566	F
G	0.562	0.551	0.557	0.559	0.555	0.539	0.559	0.563	0.561	0.546	0.543	0.562	G
H	0.565	0.594	0.591	0.595	0.593	0.586	0.572	0.576	0.591	0.57	0.598	0.569	H
	1	2	3	4	5	6	7	8	9	10	11	12	

Optical path length analysis at 0 h

…. (variation due to dispensing and measurement)

- AVE = 0.569 ± 0.013 cm
- RSD = 2.3 %

c

	1	2	3	4	5	6	7	8	9	10	11	12	
A	0.439	0.458	0.465	0.47	0.464	0.467	0.48	0.494	0.493	0.492	0.489	0.411	A
B	0.478	0.603	0.594	0.572	0.571	0.579	0.571	0.572	0.574	0.579	0.573	0.507	B
C	0.471	0.581	0.569	0.581	0.567	0.566	0.552	0.57	0.563	0.571	0.548	0.499	C
D	0.484	0.571	0.576	0.584	0.588	0.591	0.579	0.573	0.586	0.587	0.577	0.53	D
E	0.497	0.575	0.576	0.577	0.571	0.58	0.584	0.586	0.586	0.576	0.567	0.498	E
F	0.493	0.561	0.569	0.576	0.585	0.582	0.584	0.589	0.581	0.587	0.598	0.508	F
G	0.469	0.544	0.558	0.562	0.558	0.542	0.567	0.564	0.565	0.548	0.55	0.492	G
H	0.426	0.523	0.521	0.52	0.525	0.506	0.492	0.499	0.508	0.487	0.512	0.429	H
	1	2	3	4	5	6	7	8	9	10	11	12	

Optical path length analysisat 24 h

…. (variation due to including evaporation)

- AVE = 0.541 ± 0.047 cm
- RSD (24 h) = 8.7 %

d

	1	2	3	4	5	6	7	8	9	10	11	12	
A	73	81	82	82	82	83	83	83	84	84	85	74	A
B	84	103	102	101	101	100	100	100	100	100	99	87	B
C	85	101	102	102	102	102	102	102	102	103	101	89	C
D	85	101	102	102	102	102	102	103	103	102	101	90	D
E	85	100	102	101	102	102	102	102	102	102	102	89	E
F	85	101	102	102	102	102	102	102	102	102	102	90	F
G	83	99	100	101	101	101	101	100	101	100	101	88	G
H	75	88	88	87	89	86	86	87	86	85	86	75	H
	1	2	3	4	5	6	7	8	9	10	11	12	

Remained medium ratio at 24 h

- AVE = 95.0 ± 8.7 %
- RSD = 8.2 (%)

Fig. S2. Validation of medium evaporation during 24-h plate cultivation. (a) Total amount of medium evaporation estimated by weighing plate before and after cultivation. (b) Optical path length of each well at 0 h. (c) Optical path length of each well at 24 h. (d) Remained medium ratio calculated from change in optical path length.

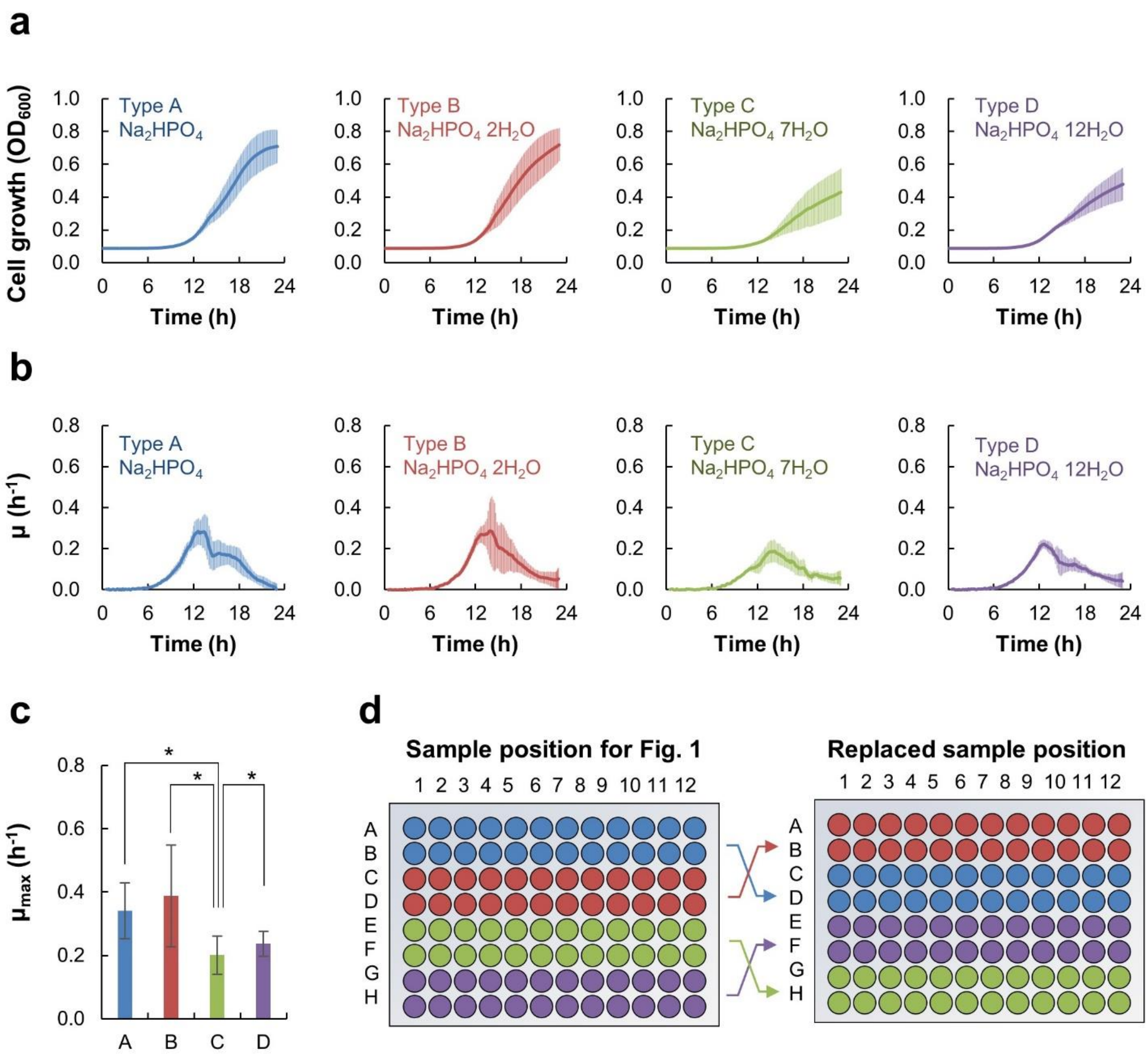

Fig. S3. Influence of difference in medium evaporation due to sample position. (a) Bacterial cell growth in M9 media (2 g/L glucose) in 96 well plates at 37 °C and 269 rpm, (b) One-hour rolling average specific cell growth rate (μ), (c) Maximum specific cell growth rate (μ_{max}), (d) Original sample position wiht the experiment for Fig. 1 and replaced sample position for this cultivation. Error bars indicate standard deviation. $*p < 0.05$ (Welch's t-test), $n = 24$.

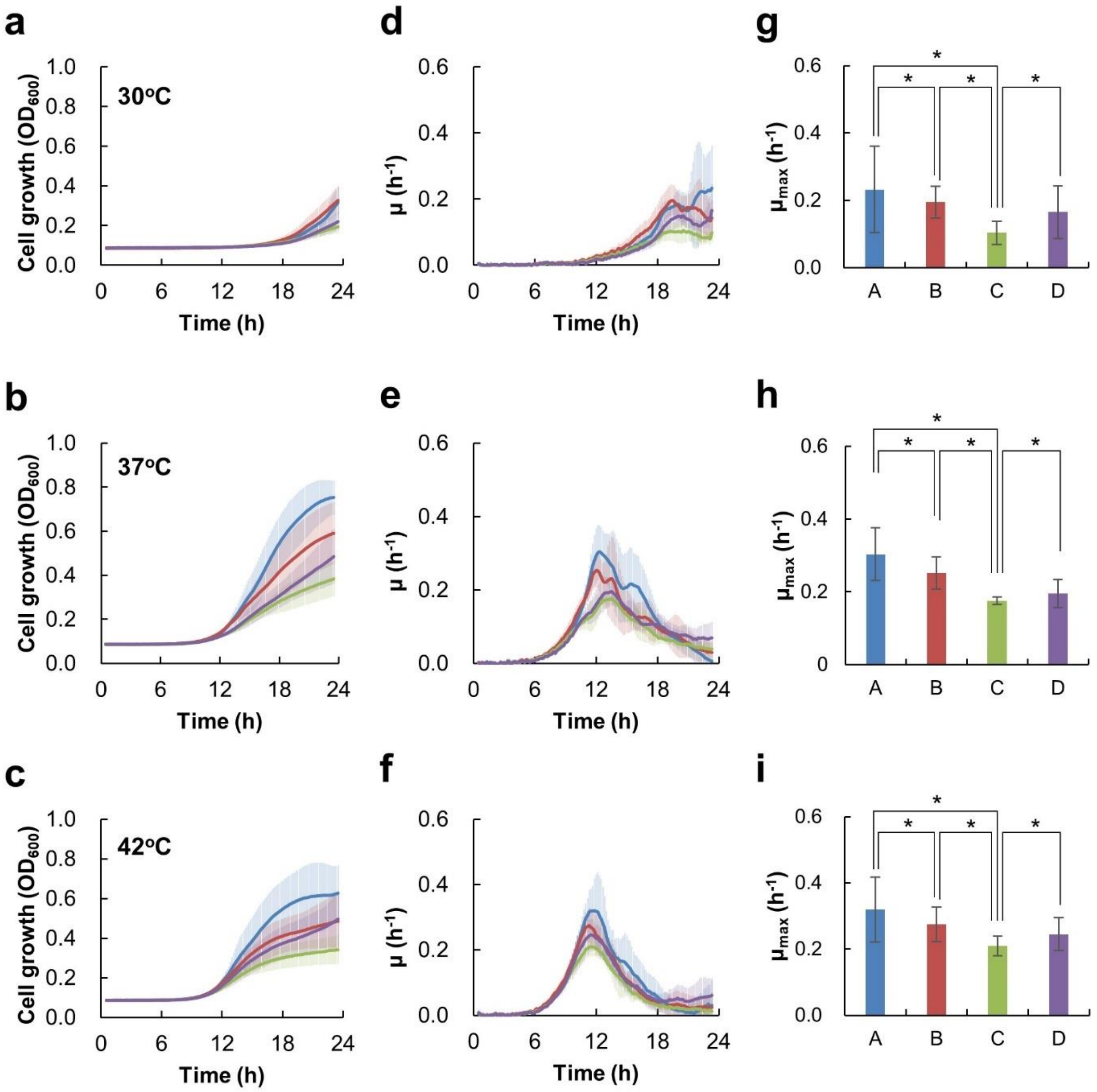

Fig. S4. Bacterial cell growth in various M9 media at different temperatures. Left panels show the cell growth curve at (a) 30 °C, (b) 37 °C, and (c) 42 °C. Middle panels show the moving average specific cell growth (μ) at (d) 30 °C, (e) 37 °C, and (f) 42 °C. Right panels show the maximum specific cell growth (μ_{max}) at (g) 30 °C, (h) 37 °C, and (i) 42 °C. Error bars indicate standard deviation. $*p < 0.05$ (Welch's t-test), $n = 24$.

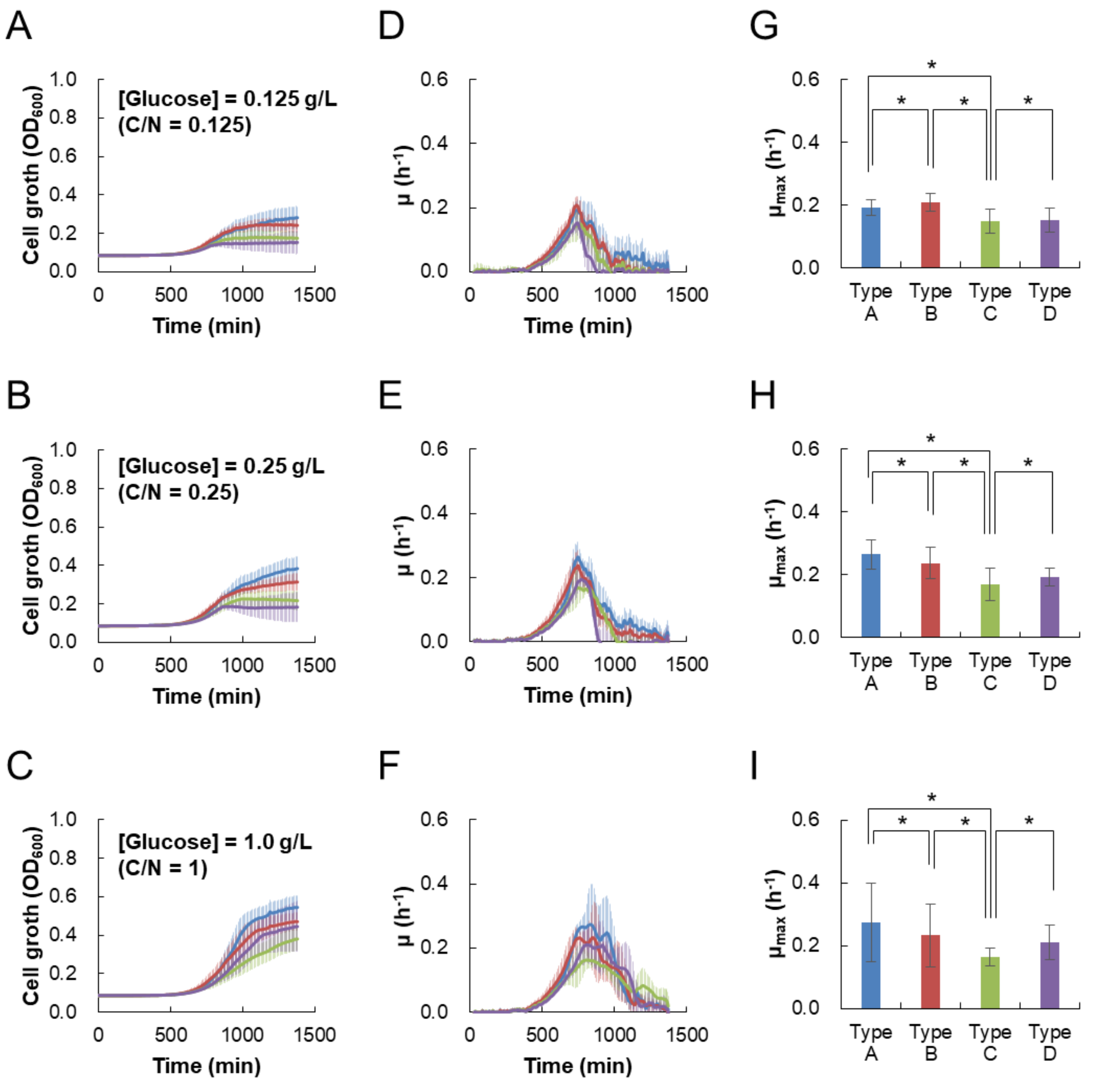

Fig. S5. Effect of phosphate hydrate type for M9 medium on bacterial cell growth of E. coli with various glucose concentration (C/N ratio). Left panels show the cell growth curve with (a) C/N=0.125, (b) C/N=0.25, and (c) C/N=1.0. Middle panels show the moving average of specific cell growth (μ) with (d) C/N=0.125, (e) C/N=0.25, and (f) C/N=1.0. Right panels show the maximum specific cell growth (μ_{max}) with (g) C/N=0.125, (h) C/N=0.25, and (i) C/N=1.0. Error bars indicate standard deviation (n=24). *$p < 0.05$ (Welch's t-test), $n = 24$.

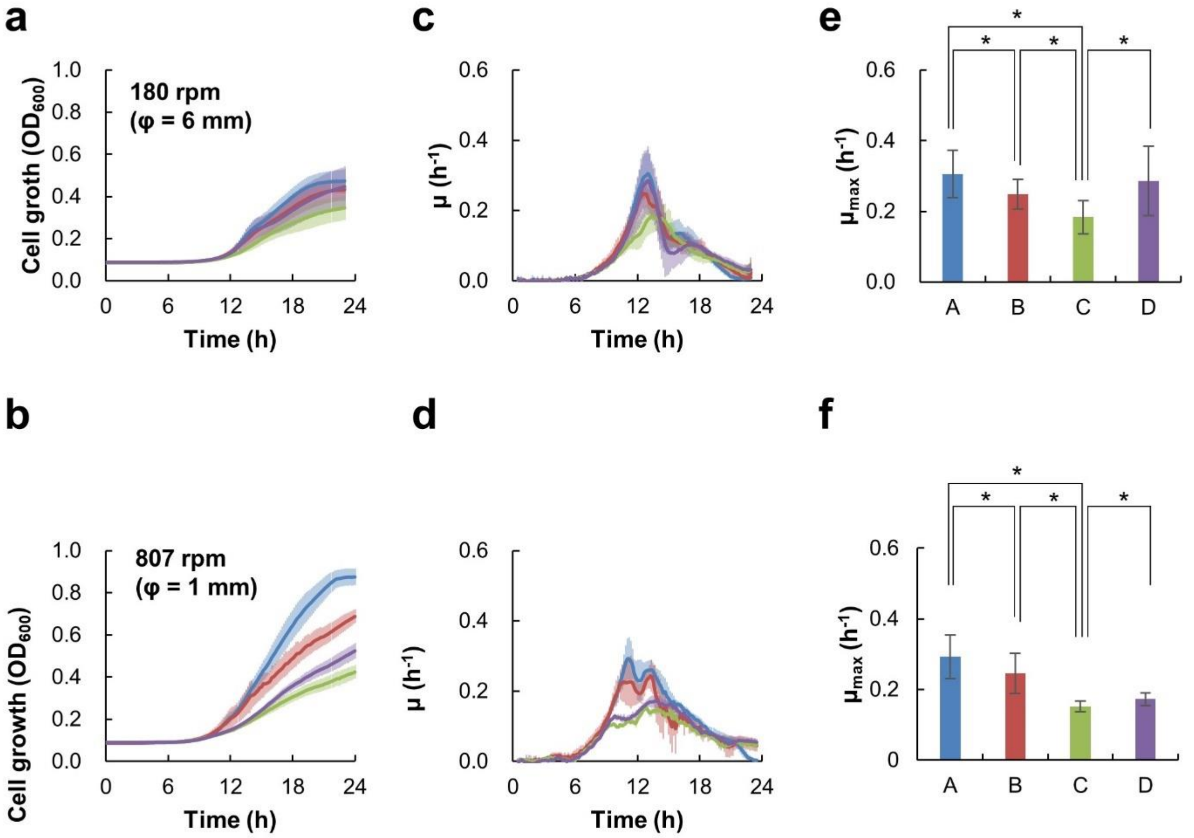

Fig. S6. Effect of phosphate hydrate type for M9 medium on bacterial cell growth of E. coli with lower (180 cpm, 6 mm) and higher (180 cpm, 6 mm) rotation shaking. Left panels show the cell growth curve with (A) lower, and (B) higher rotation shaking. Middle panels show the moving average of specific cell growth (μ) with (C) lower, and (D) higher rotation shaking. Right panels show the maximum specific cell growth (μ_{max}) with (C) lower, and (D) higher rotation shaking. Error bars indicate standard deviation (n=24). *$p < 0.05$ (Welch's *t*-test), $n = 24$.

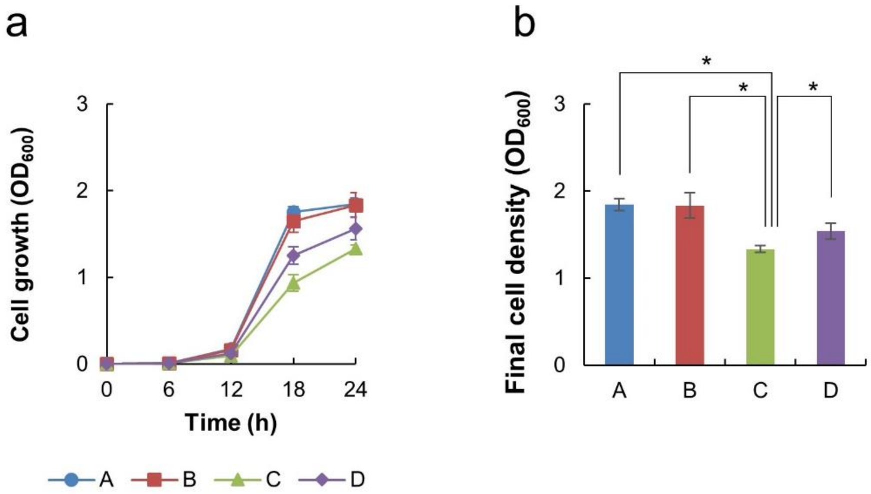

Fig. S7. Effect of phosphate hydrate type for M9 medium on bacterial cell growth of E. coli in test tube culture. (A) Cell growth curves, (B) Final cell density. Error bars indicate standard deviation (n=). *p < 0.05 (Welch's *t*-test), n = 3.

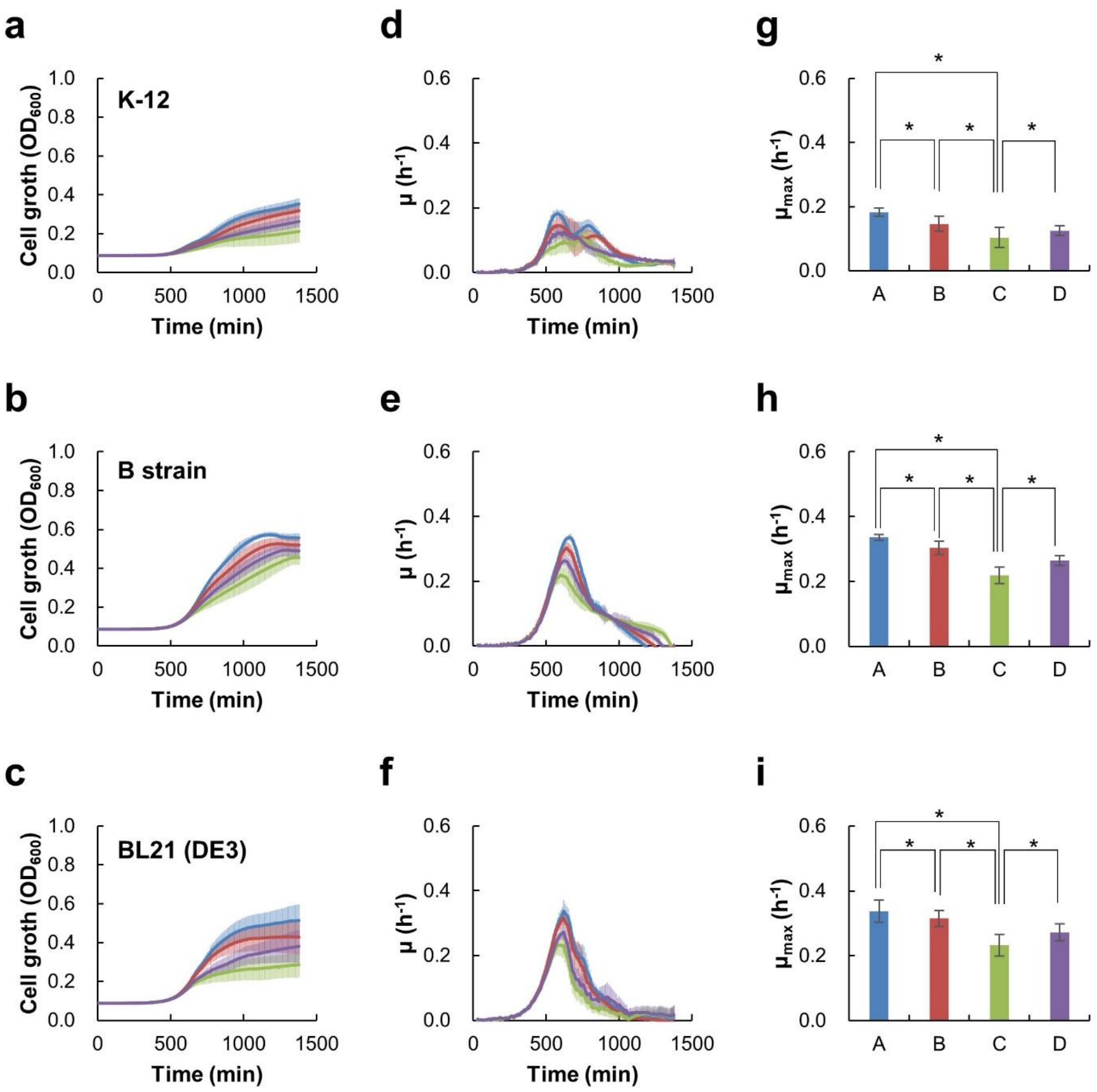

Fig. S8. Effect of M9 medium type on bacterial cell growth for different *E. coli* substrains. Left panels show the cell growth curve at (a) K-12, (b) B strain, and (c) BL21(DE3). Middle panels show the moving average of specific cell growth (μ) at (d) K-12, (e) B strain, and (f) BL21(DE3). Right panels show the maximum specific cell growth (μ_{max}) at (g) K-12, (h) B strain, and (i) BL21(DE3). Error bars indicate standard deviation. Asterisks indicate significant difference by Welch's *t*-test ($p < 0.05$), (n=24).

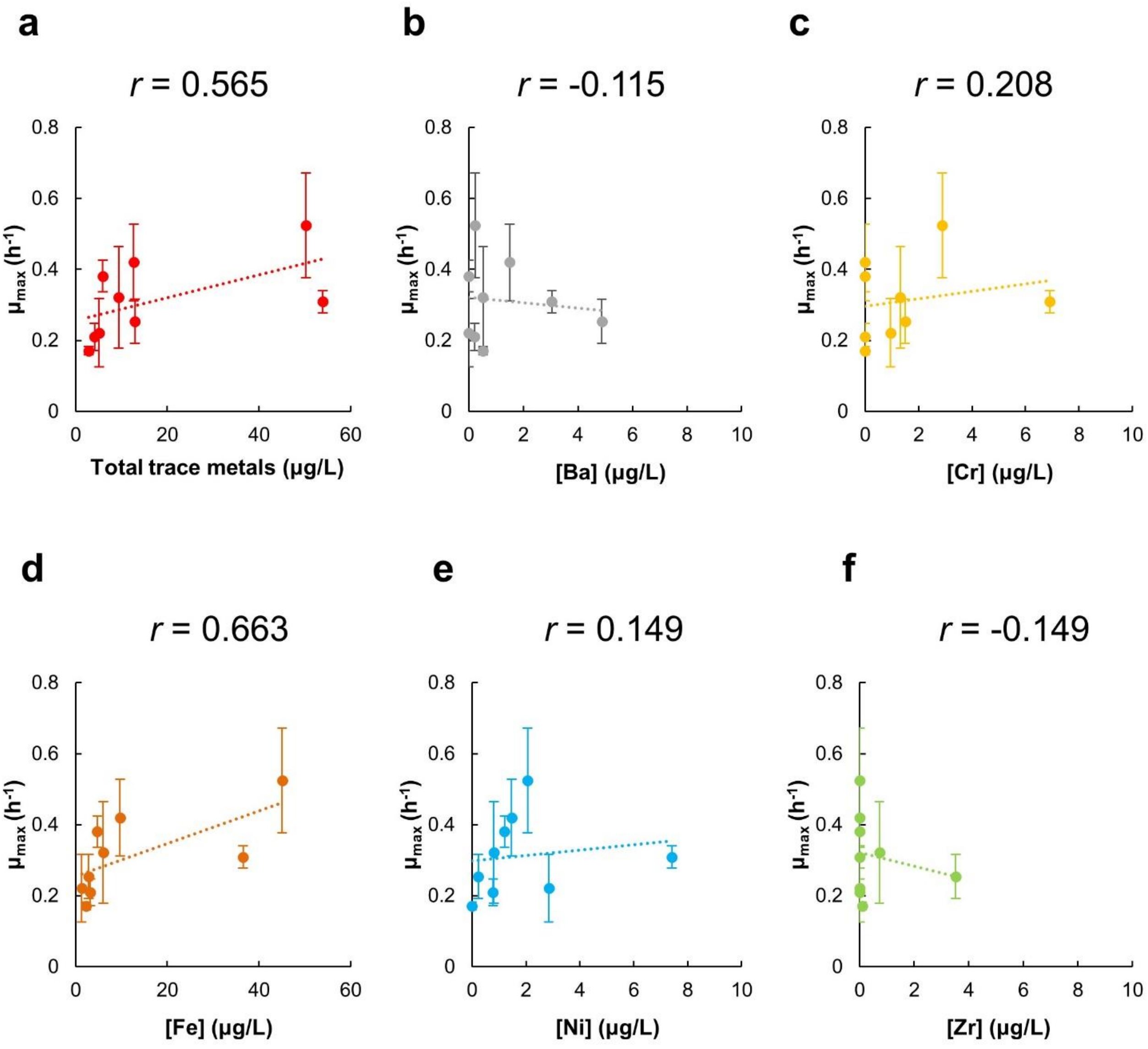

Fig. S9. Pearson correlation coefficient between trace impurity amount and the cell growth rate. Each graph shows correlation between the maximum cell growth rate (μ_{max}) and (a) total impurities amount, (b) Ba, (c) Cr, (d) Fe, (e) Ni, and (f) Zr. Error bars indicate standard deviation. Pearson's correlation coefficient *r* was showed in top of each graph.

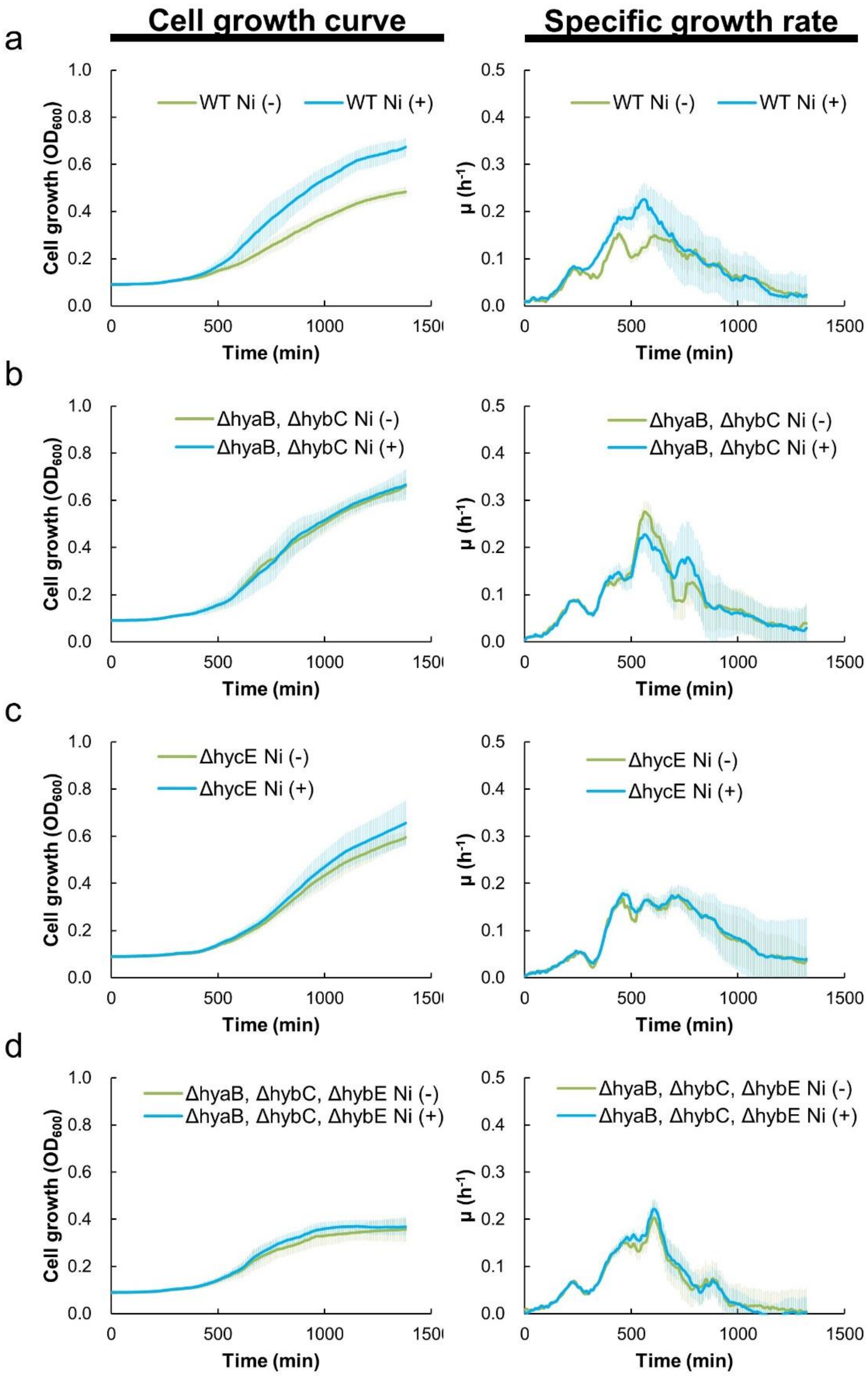

Fig. S10. Time course of cell growth and specific growth rate of [NiFe] hydrogenases deleted strains with or without Ni addition to M9 type C. (a) WT *E. coli*, (b) *ΔhyaB*, *ΔhybC* strain (c) *ΔhycE* strain (d) *ΔhyaB*, *ΔhybC hycE* strain. Left panels, cell growth curves; Right panel, 6-point moving average of specific cell growth rate (μ). Each line indicates result with M9 type C with (blue) or without (green) 0.05 μg/L of $NiSO_4$. Error bars indicate standard deviation. *$p < 0.05$ (Welch's *t*-test), $n = 8$.

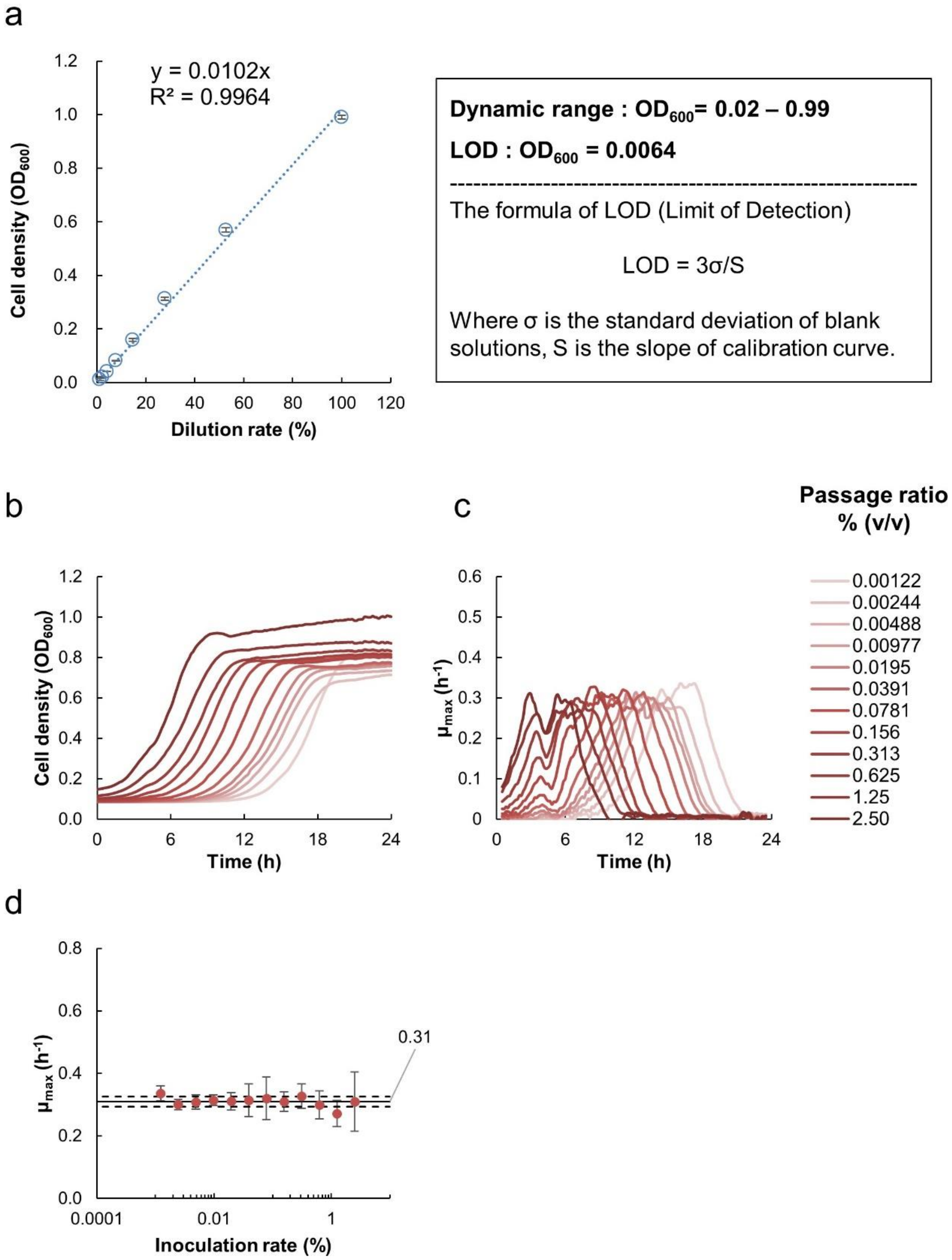

Fig. S11. Determination of initial inoculation amount for 96 well plate culture. *E. coli* strain BW25113 was passage into M9 medium from LB overnight culture with various passage ratio (0.00122 ~ 2.50 %) and cultured at 37 ºC, 250 rpm for 24 hours. (a) Measurement dynamic range of cell density using the plate reader. Correlation between the initial cell population and (b) Cell growth curves; (c) specific growth rate (μ); (d) Maximum specific growth rate (μ_{max}). Error bars indicate standard deviation (n=3).

Supplementary table

Table S1. M9 5x salts recipe from Cold Spring Harbor Protocols[†]

**M9 5x salt**		
$Na_2HPO_4•7H_2O$	64	g/L
KH_2PO_4	15	g/L
NaCl	2.5	g/L
NH_4Cl	5	g/L

Cold Spring Harbor Protocols

http://cshprotocols.cshlp.org/content/2006/1/pdb.rec614

Table S2. M9 5x salts recipe from Helmholtz Zentrum München[†]

**M9 5x salt**		
$Na_2HPO_4•2H_2O$	75.2	g/L
KH_2PO_4	30	g/L
NaCl	5	g/L
NH_4Cl	5	g/L

HelmholtzZentrumMünchen

https://www.helmholtz-muenchen.de/fileadmin/PEPF/Protocols/M9-medium_150510.pdf

Table S3. M9 5x salts recipe from SubtiWiki[†]

**M9 5x salt**		
$Na_2HPO_4•2H_2O$ (Merck)	42.5	g/L
KH_2PO_4 (Merck)	15	g/L
NH_4Cl (Merck)	5	g/L
NaCl (Merck)	2.5	g/L

SubtiWiki

http://subtiwiki.uni-goettingen.de/wiki/index.php/M9_minimal_medium

Table S4. M9 5x salts recipe from OpenWetware[†]

**M9 5x salt**		
$Na_2HPO_4•7H_2O$	64	g/L
KH_2PO_4	15	g/L
NaCl	2.5	g/L
NH_4Cl	5	g/L

OpenWetware

https://openwetware.org/wiki/M9_salts

[†] Each table shows the recipe for M9 5x salt solution which currently available publicly

Table S5. M9 type A (5x salts) ††

M9 5x salt	Recipe for 1 L		Actual value for 50 mL		Relative error	
Na_2HPO_4	33.9	g	1.695	g	0.006	%
KH_2PO_4	15	g	0.751	g	0.133	%
NaCl	2.5	g	0.125	g	0.000	%
NH_4Cl	5	g	0.252	g	0.800	%
Sterile MQ water	997	g	49.856	g	0.012	%
Total weight	1053.4	g	52.7	g	0.017	%

Table S6. M9 type B (5x salts) ††

M9 5x salt	Recipe for 1 L		Actual value for 50 mL		Relative error	
Na_2HPO_4 $2H_2O$	42.5	g	2.127	g	0.094	%
KH_2PO_4	15	g	0.751	g	0.133	%
NaCl	2.5	g	0.125	g	0.000	%
NH_4Cl	5	g	0.252	g	0.800	%
Sterile MQ water	988.4	g	49.910	g	0.992	%
Total weight	1053.4	g	53.2	g	0.940	%

Table S7. M9 type C (5x salts) ††

M9 5x salt	Recipe for 1 L		Actual value for 50 mL		Relative error	
Na_2HPO_4 $7H_2O$	64.02	g	3.208	g	0.214	%
KH_2PO_4	15	g	0.751	g	0.133	%
NaCl	2.5	g	0.125	g	0.000	%
NH_4Cl	5	g	0.252	g	0.800	%
Sterile MQ water	966.9	g	48.720	g	0.777	%
Total weight	1053.4	g	53.056	g	0.732	%

Table S8. M9 type D (5x salts) ††

M9 5x salt	Recipe for 1 L		Actual value for 50 mL		Relative error	
Na_2HPO_4 $12H_2O$	85.5	g	4.275	g	-0.039	%
KH_2PO_4	15	g	0.751	g	0.133	%
NaCl	2.5	g	0.125	g	0.000	%
NH_4Cl	5	g	0.252	g	0.800	%
Sterile MQ water	945.4	g	47.112	g	-0.332	%
Total weight	1053.4	g	52.5	g	-0.295	%

†† Each table shows the recipe of M9 5x salt and the actual used reagents amount in this study.

Table S9. Result of ICP-MS analysis

	Trace metals from disodium phosphate (µg/L in M9 medium)				
	**Ba**	**Cr**	**Fe**	**Ni**	**Zr**
A	0.72 ± 0.05	1.32 ± 0.14	5.99 ± 0.31	0.80 ± 0.07	0.72 ± 0.05
A(1G)	n.d.	2.88 ± 0.29	45.03 ± 0.39	2.06 ± 0.27	n.d.
A(GR)	n.d.	0.95 ± 0.15	1.28 ± 0.08	2.85 ± 0.28	n.d.
B	3.52 ± 0.11	1.50 ± 0.12	2.80 ± 0.18	0.23 ± 0.07	3.52 ± 0.11
C	0.09 ± 0.08	n.d.	2.22 ± 0.09	n.d.	0.09 ± 0.08
C(ACS)	n.d.	6.91 ± 0.30	36.50 ± 0.67	7.42 ± 0.20	n.d.
D	n.d.	n.d.	3.13 ± 0.45	0.78 ± 0.05	n.d.
D(1G)	n.d.	n.d.	9.66 ± 0.83	1.47 ± 0.09	n.d.
D(GR)	n.d.	n.d.	4.70 ± 0.12	1.21 ± 0.27	n.d.
